# Determinants of dietary adequacy and diet quality of in-school adolescent girls in Nigeria: implications for non-communicable diseases

**DOI:** 10.1186/s40795-026-01279-3

**Published:** 2026-02-17

**Authors:** Motunrayo Funke Olumakaiye, Gideon Onyedikachi Iheme, Patience Kehinde Alagbo

**Affiliations:** 1 Department of Human Nutrition and Dietetics, Obafemi Awolowo University, Ile-Ife, Nigeria; 2 Department of Food Studies, Nutrition and Dietetics, Uppsala University, Uppsala, SE-751 22 Sweden; 3 Department of Human Nutrition and Dietetics, Michael Okpara University of Agriculture, Umudike, Nigeria

**Keywords:** Dietary intake, Food choices, NCD-Protective foods, NCD-Risk foods, Young girls, Nigeria

## Abstract

**Background:**

An unhealthy diet is a major risk factor for non-communicable diseases (NCDs), compounded by the rising incidence of overweight/obesity among adolescent girls. This study investigated adolescent girls’ dietary adequacy and quality, their determinants and implications for NCDs.

**Methods:**

This descriptive cross-sectional study design employed a stratified random sampling technique to obtain data from a validated questionnaire and other assessments. Data was collected in 2023 from 2261 in-school adolescent girls in Nigeria. Dietary adequacy (All-5 food groups) and diet quality (NCD-Protect and NCD-Risk food groups) were assessed in accordance with global dietary recommendation (GDR). Body Mass Index-for-age (BMI) was determined using the WHO growth reference. All analyses were conducted using descriptive and inferential statistics in SPSS Version 27, with statistical significance set at *p* < 0.05.

**Results:**

Mean values recorded were age (14.9 ± 1.8), BMI (20.1 ± 3.7 kg/m²), All-5 (3.8 ± 1.1), NCD-Protect (2.4 ± 1.4), NCD-Risk (1.8 ± 1.7), and GDR (9.7 ± 1.5). About 7% were overweight/obese. Older adolescents demonstrated higher GDR (β = 0.20, *p* = 0.04) scores than their younger counterparts aged 10–13. Adolescents living in urban areas had lower diet quality than their rural counterparts (*p* < 0.001), while those from the Southeast region had higher diet quality scores. Large households showed higher All-5, NCD-Protect, and GDR scores than smaller households (*p* < 0.05). Girls who took daily money to school had higher All-5 (β = 0.03 to 0.27) but lower GDR scores (β = -0.30 to -0.22) (*p* < 0.05). An inverse association exists between All-5 (*r* = -0.043, *p* = 0.039), NCD-Protect food group (*r* = -0.054, *p* = 0.010), and BMI.

**Conclusion:**

Personal and sociodemographic characteristics influenced the respondents’ dietary adequacy and quality. Higher All-5 and NCD-Protect food group scores lowered BMI. This can positively impact overweight/obesity reduction, thereby delaying NCDs’ onset in later years. Conscious efforts should be made to communicate the potential benefits of improving adolescents’ diets.

**Supplementary Information:**

The online version contains supplementary material available at 10.1186/s40795-026-01279-3.

## Introduction

Adolescence is a critical transition phase from childhood to adulthood. It is the second-fastest period of growth after infancy and a crucial time for significant health development and well-being [1]. It is characterized by significant physical, psychological, and social changes. During this stage, adequate nutrition is essential to support rapid growth and development and establish healthy eating patterns to prevent future health issues [1].

Globally, adolescent nutrition has been a growing concern, with many adolescents experiencing both undernutrition and overnutrition. According to the Global Nutrition Report [2], the prevalence of overweight and obesity among adolescents has been increasing worldwide, with significant variations across regions [2]. More than two-thirds or one billion adolescent girls and women suffer malnutrition – undernutrition and micronutrient deficiencies [3]. South Asia and Sub-Saharan Africa are home to more than half of the malnourished young girls and women in the world [3]. The situation is quite complex in Africa, as many countries face a dual burden of malnutrition. Studies have shown that while some adolescents suffer from undernutrition, others are increasingly affected by overweight and obesity due to the nutrition transition towards more energy-dense, nutrient-poor diets [4–6]. This dual burden of malnutrition is a significant public health concern, as it compromises immunity and increases the risk of developing non-communicable diseases (NCDs) such as obesity, diabetes, nutrition-induced cancers, and cardiovascular diseases later in life [5, 7–9].

Numerous factors, such as dietary factors, influence this phase, which could profoundly affect their nutrient intake [10–12]. The assessment of dietary adequacy and diet quality is crucial for understanding the nutritional risks of adolescents and identifying areas for intervention. Previous research has highlighted the importance of consuming a balanced diet that includes a variety of food groups to ensure adequate intake of essential nutrients [13, 14]. Despite this, there is limited data on the dietary adequacy and diet quality of adolescent girls in Nigeria, particularly in relation to their risk of developing non-communicable diseases (NCDs).

During this phase, adolescents establish patterns of behaviour related to diet and the choices made can protect their health or put their health at risk now and in the future [15].

Notably, adolescent girls are particularly vulnerable, underscoring the importance of understanding and addressing their unique health needs [16].

Adolescence represents a window of opportunity to prepare nutritionally for a healthy household. It is a timely period to shape and consolidate healthy eating by preventing or postponing the onset of nutrition-related chronic diseases in adulthood. In implementing intervention programmes, it is important to investigate what foods are eaten and what determines the choice.

Therefore, this study was designed to investigate dietary adequacy, diet quality, and their determinants, focusing on the implications for non-communicable diseases. Two hypotheses were tested: (i) There is no significant association between personal and sociodemographic factors and dietary adequacy and diet quality, and (ii) There is no relationship between Dietary adequacy, diet quality and body mass index among adolescent girls in Nigeria.

## Methodology

### Study design and sampling procedure

This study was a descriptive cross-sectional study design among in-school female adolescents aged 10–19 selected from three out of the six geopolitical zones in Nigeria. Two states were selected from the three zones, namely, North Central (NC) (Kogi and Niger States), South East (SE) (Abia and Imo States) and South West (SW) (Osun and Ondo States).

Sampling was done using a multistage stratified random sampling procedure to select schools and students who participated in this study. In the first stage, six states were selected. In the second stage, the local government areas (LGAs) in each of the selected states were grouped according to their urban, peri-urban or rural status. Two most urban LGAs and two most peri-urban LGAs were selected in each state. In the third stage, the lists of secondary schools in the selected LGAs were obtained from their respective local education authorities; two schools were randomly selected from each of the LGAs. Twenty-five students each were randomly selected in the junior and senior arms of the designated schools.

School registers were used to generate a sampling framework from which eventual participants were selected using a systematic sampling technique with a K^th^ interval once a participant had been chosen randomly.

### Sample size determination

The sample size was determined using the Research Advisors [17] at 95% confidence and 5% margin of error. Since parameters were determined in each of the six selected states by LGAs, the selected LGAs from each state were treated as a population. The mean population of in-school adolescent girls in the LGAs was 12,456. According to the Research Advisor, the sample of 370 was considered sufficient to allow rational estimation in each of the six states based on the population size. Assuming a non-response rate of 5%, the adjusted calculated sample size for each of the six states was 372, and this translated to a total minimum sample size of 2,232.

### Inclusion and exclusion criteria

Adolescent girls aged 10–19 attending government-owned secondary schools were included in this study. Those who attended boarding schools, pregnant and lactating adolescents, those who lived in orphanage homes, and those who attended privately owned schools were excluded.

### Research instrument

#### Personal and sociodemographic characteristics

A validated questionnaire was used to elicit information on personal, social and demographic variables. These include exact/precise information of their age (date of birth, converted to years), household size, number of meals eaten in a day, and amount of daily money taken to school. Household size was collected by asking the adolescent girls how many people lived in the same house with them and ate at least one daily meal from the same pot. For the number of meals eaten in a day, the study participants were asked how many meals they mostly ate daily, while they were asked if they took daily money to school and the amount. A follow-up question asked what they usually spend this money on (e.g., food, drinks, snacks, transportation, other purchases). The amount spent on food, drinks and snacks was recorded.

#### Anthropometric measurements

All anthropometric data were collected using the procedures recommended by the World Health Organisation (WHO) [18]. Weight and Height measurements were determined using a 150 kg Seca Weighing Scale with a 75–200 cm stadiometer. The weight was measured without footwear and with minimal clothing, then the readings were taken to the nearest 0.1 kg. The readings were taken in triplicate, and the average was taken [18, 19]. The height was taken with the adolescent girl’s shoes removed, both feet parallel to each other. The height measurement was read to the nearest 0.01 cm. The readings were taken in triplicate, and the average was taken [19].

#### Food Frequency Questionnaire (FFQ)

An FFQ was used to collect information on the foods, drinks, and snacks consumed by the girls over seven days prior to the survey. This study instrument outlines the details of the data collected and is available in Supplementary File 1.

### Data collection

Four research assistants were recruited and trained for two days on anthropometric measurements and the use of Open Data Kit to assist in data collection for each state. The field assistants worked in pairs to reduce interpersonal bias. Each respondent was interviewed for 15 min. The data were collected concurrently for two weeks in April 2023 in all the states.

### Variables categorization

#### Personal and socio-demographic characteristics

Data on personal and socio-demographic characteristics were categorized based on pre-defined criteria and the distribution of observed values.

Some studies have classified adolescents into three stages based on the various age-related development milestones: early (10–13 years), mid (14–16), and late (17–19) [20, 21]. This was adopted to categorize the participants into age in this study. According to the Demographic Health Survey Classification, households were classified into three groups: small (< 5 members), moderately large (5–8 members) and large (> 8 members) [22].

Although there is no widely accepted consensus definition of what constitutes a regular diet, at least three meals a day alongside specific requirements have been suggested [23, 24]. This informed the classification of meal intake into one, two, three, and more than three options in this study.

Based on the observed values in this study, the amount of money taken to school was classified into simple binary groups, the daily average amount spent on food, drinks and snacks as less than 200 naira and above 200 naira.

Body Mass Index (BMI) was calculated to determine the nutritional status as the weight in kilograms divided by the square of the height in meters (kg/m^2^). It was classified using the sex-specific BMI-for-age Z-scores WHO Growth Reference for school-aged children and adolescents [25]. Percentile of <5th = Underweight, 5th -85th percentile = Healthy weight, 85th -95th percentile = Overweight and > 95th percentile = Obese. This was done using WHO AnthroPlus. This was further categorized into binary groups: non-overweight/obese and overweight/obese.

### Dietary adequacy and diet quality

Using data collected from FFQ, a dietary assessment was done using the Global Dietary Quality toolkit, which measures the adequacy and quality of diets. Our study was adapted to capture dietary intake over seven days rather than 24-hour recall. The ‘All-5’ food classification indicator was used to evaluate dietary adequacy. The total score obtainable was five if all the five food groups were consumed in the previous seven days. All-5 includes at least one vegetable, at least one fruit, at least one pulse, nut or seed, at least one animal-source food, and at least one starchy staple [26]. In addition, diet quality was assessed using ‘NCD-Protect (NCD-P)’ and ‘NCD-Risk (NCD-R)’ classification as indicators of protection against or predisposition towards noncommunicable diseases (NCDs). NCD-P consists of nine healthy food groups, with nine being the maximum score obtainable. A higher score indicates the inclusion of more health-promoting foods in the diet [26]. Likewise, NCD-R food classification also contains eight food groups; however, processed meat has a score of two as against one for others, this makes the total score obtainable to be nine. The NCD-R score is also a proxy for ultra-processed food intake. An elevated score is closely related to higher ultra-processed food consumption and higher consumption of foods and drinks to avoid or limit.

The global dietary recommendations (GDR) indicate adherence to global dietary recommendations. This is calculated as: NCD-P - NCD-*R* + 9 = GDR score. The GDR score ranges from 0 to 18 and this indicates the level of adherence to global dietary recommendations that are protective against non-communicable diseases. A high GDR score, implies that more recommendations are likely to be met [26].

### Data analysis

Verified data were entered into the computer using SPSS version 27 and analyzed using descriptive and inferential statistics. The appropriate descriptive statistics used include frequency, percentage, mean, and standard deviation, while inferences were made using multiple linear regression and Spearman’s Rho correlation analyses. The independent variables: region, residential area (urban/peri-urban), age, household size, number of daily meals consumed and amount of daily money taken to school were included in categorical forms, while the scores of the dependent variables; dietary adequacy (All-5), diet quality (NCP-P, NCD-R food groups) and GDR were incorporated in continuous forms. For the Spearman’s Rho correlation analysis, all variables in the model were in continuous form. The multiple linear regression model was subjected to the Kolmogorov-Smirnov Test for normality. The statistical significance was set to a p-value of less than 0.05. The outputs were presented using tables.

## Results

### Personal and sociodemographic characteristics of adolescent girls

Data in Table 1 indicated that proportional distribution was observed across regions, with the South West having 38.7%. Slightly more respondents resided in urban (51.7%) compared to peri-urban areas (48.3%). Over half (58.2%) were 14–16 years old, with a mean of 14.9 ± 1.8 years. Over 40% were from small and moderately large households, with an average of 6.1 ± 2.6 household members. Most of the girls (86.6%) ate three or more meals daily, and 35.3% took above 200 naira to school daily (Mean ± SD = ₦190.3 ± 35.0). The prevalence of overweight and obese was 6.9% (Mean ± SD = 20.1 ± 3.7 kg/m^2^).

**Table 1:** Personal and sociodemographic characteristics of adolescent girls (*n* = 2261)

Characteristics	*n*	%
Region		
North Central	645	28.5
South East	742	32.8
South West	874	38.7
Residential Location		
Peri-Urban	1091	48.3
Urban	1170	51.7
Age (years)		
10–13	529	23.4
14–16	1315	58.2
17–19	417	18.4
Mean ± SD = 14.9 ± 1.8		
Household size		
< 5 (Small)	953	42.1
5–8 (Moderately Large)	1021	45.2
> 8 (Large)	287	12.7
Mean ± SD = 6.1 ± 2.6		
No of meals per day		
Once	11	0.4
Twice	293	13.0
Thrice or more	1957	86.6
Mean ± SD = 2.9 ± 0.4		
Amount of daily money taken to school (Naira)		
None	538	23.8
< 200	925	40.9
₦200 and above	798	35.3
Mean ± SD = ₦190.3 ± 35.0		
Nutritional Status		
Non-Overweight/Obese	2105	93.1
Overweight/Obese	156	6.9
Mean ± SD of Body Mass Index = 20.1 ± 3.7 kg/m^2^		

### Diet adequacy and quality of adolescent girls

Data in Table 2 indicated that most adolescent girls (80% and above) consumed starchy staples, vegetables and animal source foods and about half of them consumed fruits, pulses and nuts (All-5) the previous seven days. For the NCD-P food groups, the majority of the adolescent girls (65.9% and 93.5%) consumed whole grains and other vegetables in the previous 7 days, respectively. One out of three consumed dark green leafy vegetables, none consumed vitamin A fruits, and very few consumed the other six food groups. Surprisingly, none consumed vitamin A fruits in seven days. For the NCD-R food groups, the percentage of adolescent girls who did not consume the food groups was high. A little below half consumed baked/grain-based sweets (45.1%) and other sweets food groups (43.8%).

**Table 2:** All-5, NCD-Protect, and NCD-Risk food groups of adolescent girls (*n* = 2261)

Food Groups	*N*	%	*n*	%
	Consumed		Not Consumed	
All-5				
Starchy Staples	2156	95.4	105	4.6
Vegetables	2182	96.5	79	3.5
Fruits	1149	50.8	1112	49.2
Pulses, Nuts and Seeds	1176	52.0	1085	48.0
Animal Source Food (ASF)	1872	82.8	389	17.2
NCD-Protect				
Whole Grain	1489	65.9	772	34.1
Pulses	414	18.3	1847	81.7
Nuts and Seeds	420	18.6	1841	81.4
Vitamin A Orange Vegetables	50	2.2	2211	97.8
Dark Green Vegetables (DGV)	743	32.9	1518	67.1
Other Vegetables	2115	93.5	146	6.5
Vitamin A Fruits	-	-	2261	100.0
Citrus	77	3.4	2184	96.6
Other Fruits	210	9.3	2051	90.7
NCD-Risk				
Soft Drink	96	4.2	2165	95.8
Baked/Grain-Based Sweets	1019	45.1	1242	54.9
Other Sweets	991	43.8	1270	56.2
Processed Meats^†^	250	11.1	2011	88.9
Unprocessed Meats	489	21.6	1772	78.4
Deep Fried Foods	604	26.7	1657	73.3
Noodles and Fast Food	74	3.3	2187	96.7
Ultra-Processed Salty Snacks	196	8.7	2065	91.3

“Not met” represents non-consumption of a food group in the previous 7 days

^†^Scored 2, Met” represents consumption of a food group in the previous 7 days

### Score distribution of respondents´ dietary adequacy and diet quality

As indicated in Table 3, only 30.9% consumed all five food groups; the mean score was 3.8 *±* 1.1. A score of 5 indicates minimal adherence to dietary guidelines. The results showed low mean scores for NCD-P and NCD-R. About 18% and 16% scored four and above in the NCD-P and NCD-R food groups, with mean scores of 2.4 *±* 1.4 and 1.8 *±* 1.7, respectively. The GDR score ranges from 0 to 18 and indicates adherence to global dietary recommendations, with 30.5% scoring between 11 and 14 points and a mean of 9.7 *±* 1.5.

**Table 3:** Score distribution of diet adequacy and quality (*n* = 2261)

Score	*n*	%	Mean *±* SD	Maximum rating score
All-5 (Diet Adequacy)				5
0	15	0.7	3.8 *±* 1.1	
1	45	2.0		
2	206	9.1		
3	601	26.6		
4	695	30.7		
5	699	30.9		
NCD-Protect (Diet Quality)				9
0	6	3.1	2.4 *±* 1.4	
1	4	20.0		
2	846	37.4		
3	486	21.5		
4	231	10.2		
5	100	4.4		
6	47	2.1		
7	23	1.0		
8	6	0.3		
NCD-Risk (Diet Quality)				9
0	654	28.9	1.8 *±* 1.7	
1	507	22.4		
2	462	20.4		
3	279	12.3		
4	204	9.0		
5	89	3.9		
6	34	1.5		
7	20	0.9		
8	12	0.5		
GDR				18
2–5	11	0.5	9.7 *±* 1.5	
6–10	1559	69.0		
11–14	691	30.5		

### Personal and socio-demographic determinants of diet quality and adequacy

As summarized in Table 4, the multiple linear regression analysis explored personal and sociodemographic determinants of adolescents´ dietary adequacy (All-5 food group) and diet quality (NCD-Protect, NCD-Risk, and GDR). All models were statistically significant (*p* < 0.001).

Regional difference exists across dietary adequacy and diet quality variables. When compared with North Central, the odds for All-5 (β = 0.17, *p* < 0.05) and NCD-P (β = 0.18, *p* = 0.02) scores were higher in South East, while South West had lower odds NCD-R scores (β = 0.25, *p* = 0.01). Adolescents who live in urban areas had lower likelihoods of having elevated All-5 scores (β = -0.18; p = < 0.001), NCD-protect (β = -0.20; p = < 0.001) and GDR (β = -0.33; p = < 0.001) scores than those in peri-urban locations. Late adolescents (aged 17–19 years) had higher predicted GDR scores (β = 0.20, *p* = 0.04) than their younger adolescents (11–13 years). Interestingly, the odds for higher diet quality scores were high in moderately large (All-5; β = 0.12, *p* = 0.02; GDR Score; β = 0.16, *p* = 0.02) and large (All-5; β = 0.15, p = < 0.05; NCD-Protect; β = 0.24, *p* = 0.001; GDR Score; β = 0.32, *p* = 0.002) households than small households. Additionally, adolescents who took daily money to school were more likely to have higher All-5 (β = 0.03 to 0.27, p = < 0.001) scores but decreased GDR scores (β = -0.35 to -0.42, p = < 0.001) than those who did not take money to school.

A further analysis using Spearman’s rho correlation in Table 5 shows an inverse but significant relationship between body mass index and All-5 (*r* = -0.043, *p* = 0.039) and NCD-P (*r* =-0.054, *p* = 0.010) food. The higher the All-5 and NCD-P food groups, the lower the BMI. This has positive implications on overweight/obese reduction, invariably impacting NCD onset in later years.

**Table 4:** Multiple regression analysis showing the association between personal and socio-demographic determinants and diet quality and adequacy (*n* = 2261)

Variables	All-5			NCD-Protect			NCD-Risk			GDR Score		
	B (SE)	95% CI	*p*-value	B (SE)	95% CI	*p*-value	B (SE)	95% CI	*p*-value	B (SE)	95% CI	*p*-value
(Constant)	3.47 (0.09)	(3.30, 3.64)	< 0.001	2.25 (0.11)	(2.03, 2.46)	< 0.001	1.44 (0.14)	(1.17, 1.71)	< 0.001	9.81 (0.12)	(9.57, 10.04)	< 0.001
Region												
South East	0.17 (0.06)	(0.05, 0.29)	0.05*	0.18 (0.08)	(0.03, 0.33)	0.02*	0.17 (0.10)	(-0.01, 0.36)	0.07	0.05 (0.08)	(-0.16, 0.17)	0.95
South West	0.25 (0.06)	(0.13, 0.36)	< 0.001**	0.25 (0.07)	(0.11, 0.40)	< 0.001**	0.25 (0.09)	(0.07, 0.43)	0.01**	0.02 (0.08)	(-0.16, 0.16)	0.98
Residential location												
Urban	-0.18 (0.05)	(-0.27, -0.09)	< 0.001**	-0.20 (0.06)	(-3.16, -0.09)	< 0.001**	0.01 (0.07)	(-0.14, 0.15)	0.95	-0.21 (0.06)	(-0.33, 0.09)	< 0.001**
Age (years)												
Middle (14–16)	-0.09 (0.06)	(-0.02, 0.02)	0.09	-0.09 (0.07)	(-0.23, 0.05)	0.20	-0.14 (0.09)	(-0.31, 0.03)	0.11	0.05 (0.08)	(-0.10, 0.20)	0.53
Late (17–19)	0.05 (0.07)	(-0.09, 0.19)	0.49	0.10 (0.09)	(-0.08, 0.27)	0.30	-0.10 (0.11)	(-0.33, 0.12)	0.36	0.20 (0.10)	(0.01, 0.39)	0.04*
Household size												
Moderately Large	0.12 (0.05)	(0.02, 0.21)	0.02*	0.11 (0.06)	(-0.01, 0.23)	0.07	-0.05 (0.08)	(-0.20, 0.10)	0.54	0.16 (0.07)	(0.03, 0.29)	0.02*
Large	0.15 (0.08)	(-0.01, 0.30)	0.05*	0.24 (0.09)	(0.06, 0.43)	0.001**	-0.07 (0.12)	(-0.30, 0.15)	0.53	0.32 (0.10)	(0.12, 0.51)	0.002**
Daily money taken to school (naira)												
<₦200	0.27 (0.06)	(0.15, 0.38)	< 0.001**	0.13 (0.08)	(-0.02, 0.28)	0.09	0.35 (0.09)	(0.16, 0.53)	< 0.001**	-0.22 (0.08)	(-0.38, -0.06)	0.01**
₦200 and above	0.03 (0.06)	(0.19, 0.43)	< 0.001**	0.13 (0.08)	(-0.02, 0.28)	0.09	0.42 (0.09)	(0.24, 0.61)	< 0.001**	-0.30 (0.08)	(-0.46, -0.13)	< 0.001**

Reference Categories: North Central Region, Peri-urban Residents, Early Adolescents (10–13 years), Small Household Size (< 5), No money taken to school

^**^Statistically significant at *p* < 0.01; ^*^ Statistically significant at *p* < 0.05

**Table 5:** Spearman’s Rho correlation analysis showing the relationships between body mass index and All-5, NCD-Protect, NCD-Risk food groups consumption and global dietary recommendation scores

Variables	*r*	*p*-value
All-5	-0.043	0.039*
NCD-Protect	-0.054	0.010*
NCD-Risk	-0.011	0.593
Global Dietary Recommendation	− 0.0.033	0.117

*Significant at *p* < 0.05

## Discussion

Adolescent nutrition is pivotal in shaping health outcomes and preventing non-communicable diseases (NCDs) such as heart disease, diabetes, and some cancers [27–29]. In this study, we explored dietary adequacy (All-5) and diet quality (NCD-P and NCD-R) among adolescent girls in Nigeria. Dietary adequacy refers to meeting essential nutrient requirements for overall health [26].

The All-5 index assesses the consumption of five food groups [26]. Meeting All-5 criteria ensures dietary adequacy, which is essential for preventing deficiencies and promoting well-being. In this study, the high proportion of adolescent girls who consumed starchy staples, vegetables, and ASF in the previous seven days indicated that their foods provided essential nutrients and energy. Starchy food sources such as *garri* in granular form made from cassava are typically consumed alongside groundnuts, bean cakes or bean pudding. Additionally, traditional Nigerian meals in dough form made from cassava and yam are often paired with vegetables cooked with locus beans and animal-source foods. These findings align with a study which reported that foods categorized under starchy foods, vegetables, legumes (pulses), and animal-source foods were commonly consumed by adolescents [30]. One pooled analysis of dietary pattern amongst Nigerian adolescents reported similar high consumption of starchy staples and fruits, but a contrasting low consumption level of vegetables and animal proteins in fifty-one reviewed studies [31, 32]. This discrepancy may be attributed to the methodological difference as bulk of the reviewed studies had more than five food categories which permitted the divisions of vegetables and animal products into sub-categories. High consumption of staples and vegetables is positive, as they form the foundation of a balanced diet. The high percentage of adolescent girls meeting the ASF criterion is encouraging. Animal source foods such as fish, beef, goat, egg and poultry provide essential nutrients like protein, iron, and vitamin B12, which are nutrients of importance in adolescent girls’ health. Though the quantity consumed was not investigated, the result was used as a proxy for dietary adequacy. Consuming a variety of foods ensures that adolescents receive a wide range of essential nutrients. Different food groups provide distinct vitamins, minerals, and antioxidants to support growth, immune function, and overall well-being. A varied diet is associated with lower risk factors for chronic diseases such as heart disease, diabetes, and certain cancers [33, 34].

Emerging evidence has established the connection between diet quality metrics measured as NCD-Protect and NCD-Risk and NCDs [35, 36]. NCD-P focuses on protective dietary factors that reduce the risk of NCDs. While NCD-R assesses dietary factors associated with an increased risk of NCDs. A higher NCD-P score indicates inclusion of more health-promoting foods in the diet and correlates positively with meeting global dietary recommendations [26], while a lower NCD-R score indicates exclusion of more health-damaging foods in the diet [26].

Although an Ethiopian study compared closely with our findings that while whole grains were mainly consumed, nuts, pulses and citrus recorded low intake levels, a contrasting report was found in other vegetable intake, which received low intake [37]. Quite justifiably, Nigeria reportedly has overall higher vegetable consumption levels than Ethiopia and more diverse non-leafy vegetable options [38]. In this current study, the adolescent girls scored low in NCD-P foods, which implies they did not eat enough of NCD-P foods in the study period. Possible reasons for this could be the cost and accessibility of such foods. Healthier food options such as whole grains, nuts, and fresh produce could be more expensive in urban and peri-urban areas. Limited access to such foods in certain areas could hinder adherence to dietary guidelines. Many individuals are unaware of the importance of NCD-P foods. These foods provide essential nutrients, vitamins, minerals, protein, and micronutrients, offer healthy fats and antioxidants and support digestion and overall health. Surprisingly, none of the participants consumed vitamin A fruits in seven days. These fruits are crucial for vision, immune function, and overall health. Addressing this gap is essential, as vitamin A deficiency can have serious consequences. Understanding how specific foods impact health is crucial in nutrition education.

Our study reports that adolescent girls scored low in NCD-R, which implies that they did not consume much of NCD-R food, albeit the consumption of baked/grain-based sweets, other sweets and deep-fried foods was high. These are the commonly consumed snacks that are sold as street foods and within school premises. These snacks are frequently used as meal substitutes by adolescents. Aggressive marketing and advertising of unhealthy foods influence consumer choices. Sugary snacks and fast food are often heavily promoted. Strategies to reduce consumption of NCD-R foods, especially sweets, are vital for long-term health to mitigate disease burden and adverse outcomes in adulthood.

The Global Dietary Recommendations (GDR) score, which measures adherence to dietary guidelines for a healthy diet, suggests that adolescent girls in the study area were approximately halfway towards meeting these recommendations. It is important to note that consuming healthy and protective foods alone may not suffice to prevent NCDs if the diet still includes foods classified under the NCD risk category. Emphasis should be placed on increasing the intake of NCD-P foods among adolescents to optimize health and improve health outcomes.

Age has been implicated as important sociodemographic factor in the dietary quality and adequacy of adolescents in this study. Similar to the present study, evidence from another study suggests that older adolescents were associated with elevated diet quality score [39]. Contrasting report was observed in other studies which revealed that late-adolescents had less quality of diets [40, 41].

The dynamics in the regional differences in levels of malnutrition in Nigeria remains worrisome. In agreement with our study findings, Northern Nigerian has been reported in national reviews and surveys as the prevalent region experiencing higher cases of poor diets, micronutrient deficiencies and others food related crisis among adolescents when compared with geopolitical zones in the Southern part of Nigeria [42–44].

The majority of the participants were either from small or moderately large households of eight or fewer members. Several factors can influence the positive correlation between household size and All-5 food groups and NCD-P. Smaller households may have different dynamics, affecting food availability, meal planning, and dietary choices. Smaller households might face challenges in providing balanced meals due to limited resources. However, larger households may have more diverse food preferences due to varying tastes. Shared meals within a larger family could expose adolescents to a wider range of foods. Household size can influence food security, access to diverse foods, and meal-sharing practices. Similarly, taking money to school positively correlated with All-5 and NCD-P.

Undernutrition remains a concern, affecting growth and development. Overweight and obesity are emerging concerns in developing countries as they co-exist with underweight [6]. These conditions are associated with increased NCD risk. Monitoring weight trends and promoting healthy lifestyles are essential to prevent overweight and obesity. A significant but inverse relationship between All-5, NCD-P, and BMI recorded in this study suggests that as dietary adequacy (All-5) and protective diet quality (NCD-P) improve, BMI tends to decrease. Individuals with better dietary adequacy may consume more nutrient-dense foods (e.g., fruits, vegetables, whole grains) that promote satiety and weight management. Adequate intake of essential nutrients may lead to better metabolic regulation, thereby, reducing the risk of excess weight gain.

The rising prevalence of overweight/obesity in the sampled population, which has also been reported in several studies [6, 45–48] on Nigerian adolescent girls, calls for specific interventions. For the overweight/obese girls, there is the need to educate about portion control, weight monitoring, physical exercise and mindful eating to prevent excessive weight gain.

### Strengths and limitations of the study

This study is one of the few large-scale investigations, which identified significant drivers of dietary adequacy among in-school adolescent girls in Nigeria. The application of global dietary frameworks (All-5, NCD-Protect, NCD-Risk, and GDR) alongside anthropometric measures such as BMI-for-age provides a basis for examining diet-related NCD risks in this population.

Nonetheless, the cross-sectional design limits the ability to draw causal inferences between dietary factors and health outcomes. Additionally, the use of a non-validated food frequency questionnaire (FFQ) may have introduced recall and reporting bias, potentially affecting the accuracy of dietary intake data. The reliance on self-reported information, particularly among adolescents, further compounds this limitation. Moreover, the study did not include biochemical assessments or other clinical indicators, which could have provided a more comprehensive evaluation of nutritional and NCD risk status.

## Conclusion

This study’s findings showed that the personal and sociodemographic characteristics of adolescent girls significantly determine dietary adequacy and diet quality, which also inversely correlate with body mass index. These findings have implications for preventing obesity-related NCDs in later years.

Therefore, promoting healthy eating habits during adolescence to prevent NCDs later in life is essential. This can be done through conscious efforts to communicate the benefits of improving adolescents’ diets for future healthy living. A co-created interactive nutrition education strategy that is more acceptable to adolescents, particularly girls, can be adopted.

## Supplementary Information

Supplementary Material 1.

## Data Availability

Data supporting the findings of this study are available from the corresponding author upon request.

## Abbreviations

ASF: Animal Source Food
BMI: Body Mass Index
FFQ: Food Frequency Questionnaire
GDR: Global Dietary Recommendations
GDQ: Global Dietary Quality
LGA: Local Government Area
NCD-P: NCD-Protect
NCD-R: NCD-Risk
NCDs: Non-Communicable Diseases
ODK: Open Data Kit
WHO: World Health Organisation
