# Supplementary material for: Determinants of dietary adequacy and diet quality of in-school adolescent girls in Nigeria: implications for non-communicable diseases
Source: BMC Nutr. 2026 Feb 17;12:57. doi: 10.1186/s40795-026-01279-3 (PMC13019960; doi:10.1186/s40795-026-01279-3)
Supplement: Supplementary file 1 — Supplementary Material 1. [file 40795_2026_1279_MOESM1_ESM.docx]

**SUPPLEMENTARY FILE 1**

**QUESTIONNAIRE ON THE DIETARY ADEQUACY AND DIET QUALITY** **OF FEMALE ADOLESCENTS IN NIGERIA**

Dear Respondent,

This is a study in the Department of Human Nutrition and Dietetics, Obafemi Awolowo University, Ile-Ife, Nigeria. It is meant to assess the Dietary Adequacy and Diet Quality of Female Adolescents (10-19 years) in Nigeria. I would appreciate your cooperation in filling out this survey questionnaire. All the information will be used for research purposes and kept confidential.

**IDENTIFICATION (To be completed by the Interviewer)**

**Region**: North Central [ ] South East [ ] South West [ ] ]

**Residence:** Rural [ ] Peri-Urban [ ] Urban [ ]

**Local Government Area Code**: _____________________________

**GPS Coordinates: _________________________________**

**Town/Village Name**_______________________________________________

**School Name**_________________________________________________________

**Class:** JSS 1 [ ] JSS 2 [ ] SSS 1 [ ] SSS 2 [ ]

**SECTION 1. RESPONDENT'S BACKGROUND SOCIO-DEMOGRAPHIC CHARACTERISTICS**

1. Date of Birth: Day_________ Month_____________ Year___________

2. Age _____________

3. How many people live in the household that you are living? ________________

4. Do you take money to school? Yes [ ] No [ ]

5. If yes, how much? ______________naira

6. How many times do you eat in a day? Once [ ] Twice [ ] Thrice [ ] More than thrice [ ]

**Anthropometric Measurements**

7. Weight ___________kg

8. Height ______________m

9. Body Mass Index ______________kg/m^2^

**SECTION 2: FOOD FREQUENCY**

**Please answer every question – do not leave ANY lines blank. Please tick on every line.** Over the past one week, how often did you consume or drink each of the listed foods or drinks?

| **S/N** | **Food Group** | **Not eaten** | **1day a wk** | **2days a wk** | **3days a wk** | **4days a wk** | **5days a wk** | **6days a wk** | **7days a wk** |
| --- | --- | --- | --- | --- | --- | --- | --- | --- | --- |
| 1 | Rice |  |  |  |  |  |  |  |  |
|  | Jollof rice |  |  |  |  |  |  |  |  |
|  | Fried rice |  |  |  |  |  |  |  |  |
|  | White rice |  |  |  |  |  |  |  |  |
|  | Corn/Maize |  |  |  |  |  |  |  |  |
|  | Tuwo masara |  |  |  |  |  |  |  |  |
|  | Tuwo shinkarfa |  |  |  |  |  |  |  |  |
|  | Abari |  |  |  |  |  |  |  |  |
|  | Masa |  |  |  |  |  |  |  |  |
|  | Semovita, |  |  |  |  |  |  |  |  |
|  | Pap |  |  |  |  |  |  |  |  |
|  | Roasted corn |  |  |  |  |  |  |  |  |
|  | Cocopops |  |  |  |  |  |  |  |  |
|  | Custard |  |  |  |  |  |  |  |  |
|  | Cornflakes, |  |  |  |  |  |  |  |  |
|  | Golden morn, |  |  |  |  |  |  |  |  |
|  | Wheat |  |  |  |  |  |  |  |  |
|  | Quakers oat |  |  |  |  |  |  |  |  |
|  | Wheat Porridge |  |  |  |  |  |  |  |  |
|  | Bread |  |  |  |  |  |  |  |  |
|  | Sorghum, Millet |  |  |  |  |  |  |  |  |
|  | Noodles |  |  |  |  |  |  |  |  |
|  | Couscous |  |  |  |  |  |  |  |  |
|  | Pasta (Spaghetti/Macaroni) |  |  |  |  |  |  |  |  |
| 2. | **Roots, Tubers and Plantain** |  |  |  |  |  |  |  |  |
|  | Potatoes (Irish/Sweet) |  |  |  |  |  |  |  |  |
|  | Yam Roasted.Bolied/Fried) |  |  |  |  |  |  |  |  |
|  | Yam porridge |  |  |  |  |  |  |  |  |
|  | Cassava |  |  |  |  |  |  |  |  |
|  | Cassava flakes (Gaari/Eba) |  |  |  |  |  |  |  |  |
|  | Fufu |  |  |  |  |  |  |  |  |
|  | Starch |  |  |  |  |  |  |  |  |
|  | Abacha |  |  |  |  |  |  |  |  |
|  | Cocoyam |  |  |  |  |  |  |  |  |
|  | Cocoyam porridge |  |  |  |  |  |  |  |  |
|  | Plantain (Boiled/Fried) |  |  |  |  |  |  |  |  |
|  | Pear (Ube) |  |  |  |  |  |  |  |  |
| 3. | **Vitamin A rich Vegetables and Tubers** |  |  |  |  |  |  |  |  |
|  | Carrot, |  |  |  |  |  |  |  |  |
|  | Red or orange fleshed sweet potato |  |  |  |  |  |  |  |  |
|  | Beetroot |  |  |  |  |  |  |  |  |
|  | Red sorrel leaf-Zobo |  |  |  |  |  |  |  |  |
| 4. | **Dark green leafy vegetables DGLV** |  |  |  |  |  |  |  |  |
|  | Amaranth (Efo tete) |  |  |  |  |  |  |  |  |
|  | Cassava leaves |  |  |  |  |  |  |  |  |
|  | Pumpkin leaf (elegede), Yakwa leaf-Miyan Taushe, |  |  |  |  |  |  |  |  |
|  | Kale (Efo Igbo/igba) |  |  |  |  |  |  |  |  |
|  | Spinach |  |  |  |  |  |  |  |  |
|  | Ugu |  |  |  |  |  |  |  |  |
|  | Bitterleaf |  |  |  |  |  |  |  |  |
|  | Waterleaf |  |  |  |  |  |  |  |  |
|  | Bushbuck-Utazi leaf |  |  |  |  |  |  |  |  |
|  | Bitter melon leaf |  |  |  |  |  |  |  |  |
|  | Achara leaf (Ofe achara soup) |  |  |  |  |  |  |  |  |
|  | Jute leaves-Ewedu (Kerenkere) |  |  |  |  |  |  |  |  |
|  | African eggplant leaf (Igbagba) |  |  |  |  |  |  |  |  |
| 5. | **Other Vegetables** |  |  |  |  |  |  |  |  |
|  | Okro |  |  |  |  |  |  |  |  |
|  | Cabbage |  |  |  |  |  |  |  |  |
|  | Lettuce |  |  |  |  |  |  |  |  |
|  | Garlic |  |  |  |  |  |  |  |  |
|  | Cauliflower |  |  |  |  |  |  |  |  |
|  | Cucumber |  |  |  |  |  |  |  |  |
|  | Onion |  |  |  |  |  |  |  |  |
|  | Eggplant |  |  |  |  |  |  |  |  |
|  | Tomato |  |  |  |  |  |  |  |  |
|  | Red pepper |  |  |  |  |  |  |  |  |
|  | Mushroom |  |  |  |  |  |  |  |  |
|  | Green beans |  |  |  |  |  |  |  |  |
|  | Green Pepper |  |  |  |  |  |  |  |  |
|  | Spring onion |  |  |  |  |  |  |  |  |
| 6. | **Vitamin A rich Fruits** |  |  |  |  |  |  |  |  |
|  | Mango |  |  |  |  |  |  |  |  |
|  | Pawpaw |  |  |  |  |  |  |  |  |
|  | Apricot |  |  |  |  |  |  |  |  |
|  | Cantaloupe melon |  |  |  |  |  |  |  |  |
| 7. | **Other Fruits** |  |  |  |  |  |  |  |  |
|  | Apple |  |  |  |  |  |  |  |  |
|  | Banana |  |  |  |  |  |  |  |  |
|  | Lemon |  |  |  |  |  |  |  |  |
|  | Berries |  |  |  |  |  |  |  |  |
|  | Pomegranate |  |  |  |  |  |  |  |  |
|  | Strawberries |  |  |  |  |  |  |  |  |
|  | Yellow Cherry |  |  |  |  |  |  |  |  |
|  | Plum |  |  |  |  |  |  |  |  |
|  | Grapefruit |  |  |  |  |  |  |  |  |
|  | Pineapple, |  |  |  |  |  |  |  |  |
|  | Guava |  |  |  |  |  |  |  |  |
|  | Pear |  |  |  |  |  |  |  |  |
|  | African Star apple (agbalumo) |  |  |  |  |  |  |  |  |
|  | Watermelon |  |  |  |  |  |  |  |  |
|  | Cashew |  |  |  |  |  |  |  |  |
|  | Oranges |  |  |  |  |  |  |  |  |
|  | Breadfruit |  |  |  |  |  |  |  |  |
|  | Soursop |  |  |  |  |  |  |  |  |
|  | Coconut |  |  |  |  |  |  |  |  |
|  | Sugarcane |  |  |  |  |  |  |  |  |
| 8. | **Flesh Foods and Organ meat** |  |  |  |  |  |  |  |  |
|  | Liver |  |  |  |  |  |  |  |  |
|  | Kidney |  |  |  |  |  |  |  |  |
|  | Heart |  |  |  |  |  |  |  |  |
|  | Beef |  |  |  |  |  |  |  |  |
|  | Cornbeef |  |  |  |  |  |  |  |  |
|  | Pork |  |  |  |  |  |  |  |  |
|  | Lamb |  |  |  |  |  |  |  |  |
|  | Goat |  |  |  |  |  |  |  |  |
|  | Rabbit |  |  |  |  |  |  |  |  |
|  | Chicken |  |  |  |  |  |  |  |  |
|  | Duck |  |  |  |  |  |  |  |  |
|  | Edible Insects |  |  |  |  |  |  |  |  |
|  | Lungs |  |  |  |  |  |  |  |  |
|  | Mutton |  |  |  |  |  |  |  |  |
|  | Turkey |  |  |  |  |  |  |  |  |
|  | Quail |  |  |  |  |  |  |  |  |
| 9. | **Eggs** |  |  |  |  |  |  |  |  |
|  | Egg from Chicken |  |  |  |  |  |  |  |  |
|  | Duck |  |  |  |  |  |  |  |  |
|  | Guinea fowl |  |  |  |  |  |  |  |  |
|  | Quail |  |  |  |  |  |  |  |  |
|  | Boiled |  |  |  |  |  |  |  |  |
|  | Fried |  |  |  |  |  |  |  |  |
|  | Scrambled |  |  |  |  |  |  |  |  |
|  | Poached |  |  |  |  |  |  |  |  |
|  | Raw |  |  |  |  |  |  |  |  |
| 10. | **Fish and Sea Food** |  |  |  |  |  |  |  |  |
|  | Fresh |  |  |  |  |  |  |  |  |
|  | Dried Fish |  |  |  |  |  |  |  |  |
|  | Shellfish |  |  |  |  |  |  |  |  |
|  | Can fish (Sardine) |  |  |  |  |  |  |  |  |
|  | Smoked fish |  |  |  |  |  |  |  |  |
|  | Cab stick |  |  |  |  |  |  |  |  |
|  | Periwinkle |  |  |  |  |  |  |  |  |
| 11. | **Legumes Nuts, and Seeds** |  |  |  |  |  |  |  |  |
|  | Sesame seed |  |  |  |  |  |  |  |  |
|  | Almonds |  |  |  |  |  |  |  |  |
|  | Pumpkin seed |  |  |  |  |  |  |  |  |
|  | Walnut |  |  |  |  |  |  |  |  |
|  | Sunflower seed |  |  |  |  |  |  |  |  |
|  | Hazelnuts |  |  |  |  |  |  |  |  |
|  | Apricot |  |  |  |  |  |  |  |  |
|  | Peanut |  |  |  |  |  |  |  |  |
|  | Locust beans |  |  |  |  |  |  |  |  |
|  | Cashew seed |  |  |  |  |  |  |  |  |
|  | Bambara (Okpa) Melon |  |  |  |  |  |  |  |  |
|  | Groundnut |  |  |  |  |  |  |  |  |
| 12. | **Beans and Peas** |  |  |  |  |  |  |  |  |
|  | Beans |  |  |  |  |  |  |  |  |
|  | Beans porridge |  |  |  |  |  |  |  |  |
|  | Moinmoin |  |  |  |  |  |  |  |  |
|  | Akara |  |  |  |  |  |  |  |  |
|  | Lentils |  |  |  |  |  |  |  |  |
|  | Chickpeas |  |  |  |  |  |  |  |  |
|  | Mung bean |  |  |  |  |  |  |  |  |
|  | Red beans |  |  |  |  |  |  |  |  |
|  | White beans |  |  |  |  |  |  |  |  |
|  | Split peas |  |  |  |  |  |  |  |  |
| 13. | **Milk and Milk Products** |  |  |  |  |  |  |  |  |
|  | Milk |  |  |  |  |  |  |  |  |
|  | Cheese (Wara) |  |  |  |  |  |  |  |  |
|  | Yoghurt |  |  |  |  |  |  |  |  |
|  | Ice cream |  |  |  |  |  |  |  |  |
|  | Condensed milk |  |  |  |  |  |  |  |  |
|  | Skimmed milk |  |  |  |  |  |  |  |  |
|  | Goat milk |  |  |  |  |  |  |  |  |
|  | Tofu |  |  |  |  |  |  |  |  |
| 14. | **Oil and Fats** |  |  |  |  |  |  |  |  |
|  | Vegetable oil |  |  |  |  |  |  |  |  |
|  | Palm oil |  |  |  |  |  |  |  |  |
|  | Butter, added to food or used in cooking |  |  |  |  |  |  |  |  |
|  | Mayonnaise |  |  |  |  |  |  |  |  |
|  | Shortenings |  |  |  |  |  |  |  |  |
|  | Margarine |  |  |  |  |  |  |  |  |
|  | Crisco |  |  |  |  |  |  |  |  |
| 15. | **Sweets** |  |  |  |  |  |  |  |  |
|  | Sugar |  |  |  |  |  |  |  |  |
|  | Honey |  |  |  |  |  |  |  |  |
|  | Sweetened Soda |  |  |  |  |  |  |  |  |
|  | Chocolates, |  |  |  |  |  |  |  |  |
|  | Candies |  |  |  |  |  |  |  |  |
| 16. | **Snacks (Flour)** |  |  |  |  |  |  |  |  |
|  | Pie |  |  |  |  |  |  |  |  |
|  | Doughnut |  |  |  |  |  |  |  |  |
|  | Chinchin |  |  |  |  |  |  |  |  |
|  | Puffpuff |  |  |  |  |  |  |  |  |
|  | Sharwama |  |  |  |  |  |  |  |  |
|  | Springroll |  |  |  |  |  |  |  |  |
|  | Buns |  |  |  |  |  |  |  |  |
|  | Biscuits |  |  |  |  |  |  |  |  |
|  | Cakes |  |  |  |  |  |  |  |  |
|  | Cookies |  |  |  |  |  |  |  |  |
|  | Kulikuli |  |  |  |  |  |  |  |  |
|  | Robo |  |  |  |  |  |  |  |  |
|  | Donkwa |  |  |  |  |  |  |  |  |
|  | Dodo Ikire |  |  |  |  |  |  |  |  |
|  | Coconut candies |  |  |  |  |  |  |  |  |
|  | Plantain chips |  |  |  |  |  |  |  |  |
|  | Cocoyam chips |  |  |  |  |  |  |  |  |
|  | Popcorn |  |  |  |  |  |  |  |  |

**CLASSIFICATION OF FOOD GROUPS**

| **Food Groups** | **Yes** | **No** |
| --- | --- | --- |
| **All-5** |  |  |
| Starchy Staples |  |  |
| Vegetables |  |  |
| Fruits |  |  |
| Pulses, Nuts and Seeds |  |  |
| Animal Source Food (ASF) |  |  |
| **NCD-Protect** |  |  |
| Whole Grain |  |  |
| Pulses |  |  |
| Nuts and Seeds |  |  |
| Vitamin A Orange Vegetables |  |  |
| Dark Green Vegetables (DGV) |  |  |
| Other Vegetables |  |  |
| Vitamin A Fruits |  |  |
| Citrus |  |  |
| Other Fruits |  |  |
| **NCD-Risk** |  |  |
| Soft Drink |  |  |
| Baked/Grain-Based Sweets |  |  |
| Other Sweets |  |  |
| Processed Meats**^†^** |  |  |
| Unprocessed Meats |  |  |
| Deep Fried Foods |  |  |
| Noodles and Fast Food |  |  |
| Ultra-Processed Salty Snacks |  |  |

**^†^** Scored 2
